# Resveratrol Prevents Right Ventricle Dysfunction, Calcium Mishandling, and Energetic Failure via SIRT3 Stimulation in Pulmonary Arterial Hypertension

**DOI:** 10.1155/2021/9912434

**Published:** 2021-06-20

**Authors:** Judith Bernal-Ramírez, Christian Silva-Platas, Carlos Jerjes-Sánchez, Martín R. Ramos-González, Eduardo Vázquez-Garza, Héctor Chapoy-Villanueva, Alicia Ramírez-Rivera, Ángel Zarain-Herzberg, Noemi García, Gerardo García-Rivas

**Affiliations:** ^1^Tecnológico de Monterrey, Escuela de Medicina y Ciencias de la Salud, Cátedra de Cardiología. N. L, Monterrey, Mexico; ^2^Tecnológico de Monterrey, Centro de Investigación Biomédica, Hospital Zambrano Hellion, San Pedro Garza García, Mexico; ^3^Unidad de Investigación Clínica en Medicina, Monterrey, Mexico; ^4^Facultad de Medicina, Universidad Nacional Autónoma de México, Departamento de Bioquímica, Ciudad de México, Mexico; ^5^Tecnológico de Monterrey, Centro de Medicina Funcional, Hospital Zambrano Hellion, San Pedro Garza García, Mexico

## Abstract

Pulmonary arterial hypertension (PAH) is characterized by pulmonary vessel remodeling; however, its severity and impact on survival depend on right ventricular (RV) failure. Resveratrol (RES), a polyphenol found in red wine, exhibits cardioprotective effects on RV dysfunction in PAH. However, most literature has focused on RES protective effect on lung vasculature; recent finding indicates that RES has a cardioprotective effect independent of pulmonary arterial pressure on RV dysfunction, although the underlying mechanism in RV has not been determined. Therefore, this study is aimed at evaluating sirtuin-3 (SIRT3) modulation by RES in RV using a monocrotaline- (MC-) induced PAH rat model. Myocyte function was evaluated by confocal microscopy as cell contractility, calcium signaling, and mitochondrial membrane potential (ΔΨ*m*); cell energetics was assessed by high-resolution respirometry, and western blot and immunoprecipitation evaluated posttranslational modifications. PAH significantly affects mitochondrial function in RV; PAH is prone to mitochondrial permeability transition pore (mPTP) opening, thus decreasing the mitochondrial membrane potential. The compromised cellular energetics affects cardiomyocyte function by decreasing sarco-endoplasmic reticulum Ca^2+^-ATPase (SERCA) activity and delaying myofilament unbinding, disrupting cell relaxation. RES partially protects mitochondrial integrity by deacetylating cyclophilin-D, a critical component of the mPTP, increasing SIRT3 expression and activity and preventing mPTP opening. The preserved energetic capability rescues cell relaxation by maintaining SERCA activity. Avoiding Ca^2+^ transient and cell contractility mismatch by preserving mitochondrial function describes, for the first time, impairment in excitation-contraction-energetics coupling in RV failure. These results highlight the importance of mitochondrial energetics and mPTP in PAH.

## 1. Introduction

Pulmonary arterial hypertension (PAH) is a complex disease resulted from the interplay of several biological and environmental processes leading to pulmonary vasculature remodeling, therefore pulmonary hypertension [1]. Consequently, the low-pressure, thin-walled, crescent-shaped RV has to overcome structural changes to accomplish its function and pump against such an increased afterload [2]. Therefore, RV hypertrophy is a necessary adaptation to preserve RV-pulmonary arterial coupling by decreasing RV wall tension and increasing RV cardiomyocyte force-generating capacity [2]. Consequently, in early stages, it emerges as an adaptative remodeling, while at end stage of the disease, it becomes a maladaptive remodeling [2]. Despite the publication of 41 randomized clinical trials in the past 25 years and the regulatory approval of multiple drugs delivered by four administration routes [3], there is no drug focused on improving RV performance and/or reducing inflammation [4]. Despite currently available therapies PAH patients remain significant morbidity and mortality [1]. A polyphenol from the stilbene family, 3,5,4′-trihydroxystilbene resveratrol (RES), has drawn researchers' attention by its cardioprotective activity in other cardiovascular diseases [5]. Although RES acts as a pleiotropic agent in several conditions, it has an intrinsic antioxidant capacity, as well as an ability to regulate membrane receptors, kinases, and other enzymes [6–8]. In PAH models, RES improves lung functioning through its antiproliferative [9], antioxidant [10], and anti-inflammatory properties [11]. More precisely, RES activates sirtuins, a relevant group of deacetylases that participate in the regulation of numerous cellular processes [12]. In the heart, sirtuin activation has been linked to the prevention of hypertrophy [13] and energetic dysfunction [14, 15].

Previously, we found the prevention of RV hypertrophy and cardiac fibrosis by RES in PAH, accompanied by a decrease in the RV acetylation profile [16]. However, the connection between these two mechanisms remains unclear. Progression to RV failure has been linked to mitochondrial dysfunction [17, 18], as PAH generates a disruption in the mitochondrial structure [17], decreasing its oxidative capability [19] and diminishing ATP production [17, 20]. The compromised cardiac energetics impairs RV contractility by reducing creatine kinase expression [21], a key component in transferring energy to myofilaments. Thus, protecting mitochondrial function with cyclosporine A (CsA), which blocks mPTP opening by interacting with cyclophilin D (CypD), prevents mitochondrial disruption in PAH and preserves RV function [22]. CypD hyperacetylation is an essential trigger of mPTP opening [23], and its contribution to mitochondrial dysfunction and heart failure has been established in animal models [24] and humans [15]. Notably, CypD acetylation is regulated by SIRT3 [23, 24], a sirtuin stimulated by RES [12], which is associated with the loss-of-function polymorphism found in PAH patients [25].

The search for additional therapeutic options for more effective PAH management has led to the development and approval of new drugs [26]; however, the available treatments focus on exclusively in managing pulmonary alterations [27]. Gaining a basic understanding of RV alterations through dysfunction, RV failure, and mechanisms that delay these changes may be helpful in developing new therapeutic strategies to improve PAH prognosis. Therefore, the aim of this study is to evaluate the extent of SIRT3 activation in the cardioprotection conferred by RES in the RV of a MC-induced PAH model.

## 2. Materials and Methods

### 2.1. Reagents

All reagents were purchased from Sigma-Aldrich (St. Louis, MO, USA), unless otherwise stated.

### 2.2. Murine Model of Pulmonary Arterial Hypertension

PAH was induced in male Sprague–Dawley rats (Bioinvert, Edo. de México, MX) weighing >300 g by a single MC (PHL8925) dose (60 mg/kg, subcutaneous) diluted in dimethylsulfoxide (DMSO, 472301), as previously reported [16]. The control group was treated with equivalent volume of DMSO. Animals were kept at 25°C with 12 h light/dark cycle. Water and food were given ad libitum. A group of MC-injected animals was treated with RES dissolved in water (20 mg/kg/day, intra gastric) during day 1 to 42 after MC injection (PAH-RES) [16]. The other groups were given equivalent volume of water (intragastric). All animals were observed for general appearance and respiratory symptomatology. A group of control animals treated with RES during 42 days (20 mg/kg/day, intragastric) was evaluated.

### 2.3. Histologic Preparations

As reported previously [16], after injection of sodium heparin (1000 U/kg), animals were anesthetized with 5% sevoflurane and the heart and lungs were removed to be fixed in 4% (wt/vol) paraformaldehyde in PBS at room temperature for more than 2 hours. Afterwards, tissues were embedded in paraffin and stained with hematoxylin/eosin (H&E) and Masson's trichrome. An Imager Z1 Zeiss microscope with an AxioCam HRm was used, and images were processed with the AxioVision software. Micrograph from the whole Masson's trichrome stained slides was taken at 2.5x; then, the image was decomposed in more than 7 fields at 5x. Fibrotic index was assessed by quantification of blue and red pixels, using ImageJ (http://imagej.nih.gov/ij/, NIH, Bethesda, MD, USA); a blue%/red% ratio was made. Two blinded analysts performed the analysis, and three different fields were analyzed. H&E micrograph from the papillary muscles was used to quantify cardiomyocyte area at 10x. An object carrier with a capacity for 7 slides was used to analyze all slides with their respective batches. Arterioles with smooth muscular medial layer proliferation were quantified in seven random fields of lung H&E micrograph, to analyze its diameter, and the occlusion vessel of 100 *μ*m was selected. Occlusion was obtained by averaging more than seven measurements of the medial layer thickness.

### 2.4. Cardiomyocyte Isolation

Ventricular myocytes were isolated modifying a previous report [28]. Briefly after, animals were heparinized (1000 U/kg, intraperitoneal) and anesthetized with sevoflurane (1.5%-3%/1 L/min, inhaled). Hearts were excised and mounted on a Langendorff apparatus to be perfused with Tyrode solution (Ty) (mM: 128 NaCl, 0.4 NaH_2_PO_4_, 6 glucose, 5.4 KCl, 0.5 MgCl-6H_2_O, 5 creatinine, 5 taurine and 25 HEPES, pH 7.4) at 37°C for 5 min and digested by collagenase type II (0.1% in Ty) (Worthington Biochemical, Lakewood, NJ). Subsequently, RV was dissected and cells were mechanically disaggregated. Cardiomyocytes were rinsed with 0.1% albumin in Ty solution at increasing Ca^2+^ concentrations (0.25, 0.5, and 1 mM). All the confocal measurements were acquired using a Leica TCS SP5 confocal microscope equipped with a D-apochromatic 63X, 1.2 NA, oil objective (Leica Microsystems, Wetzlar, Germany). Only rod-shaped cells with visible striations were selected for the study. All records were analyzed using ImageJ (http://imagej.nih.gov/ij/, NIH, Bethesda, MD, USA).

### 2.5. Cell Shortening and Ca^2+^ Handling in Intact Cardiomyocytes

Cell shortening was evaluated in 0.5 Hz paced Ca^2+^ transient records, where the scanned line was longer than the cell length, following a previous report [29]. Briefly, a rectangular region comprising both cellular edges was selected and a threshold was set to distinguish the intracellular from the extracellular space, converting the image into binary. The cell border of the resulting binary image was compared to the border on the original record to ensure a close fit. Cell shortening parameters evaluated were time to peak shortening from fully cell rest length and time to 50% of relaxation from maximal shortening. ImageJ software (http://imagej.nih.gov/ij/, NIH, Bethesda, MD, USA) was used to process images. Intracellular Ca^2+^ signaling was measured as previously reported [28, 30]. Recently isolated cardiomyocytes were incubated with 10 *μ*M Fluo-4 AM (F14201, Life Technologies, USA) (in Ty, 1 mM Ca^2+^) for 45 min at 25°C. Later, cells were washed with fluorophore-free solution, plated on laminin- (L2020) covered glass coverslips, and mounted in a superfusion chamber. Line scan images were recorded by the cell longitudinal axis (400 Hz, 1 *μ*m section thickness) by confocal microscopy under 0.5 Hz field stimulation (MYP100 MyoPacer Field Stimulator; Ion-Optix, Milton, MA, USA). Fluorophore excitation was 488 nm, and emission window was 500-600 nm. *β*-Adrenergic stimulation was assessed after 10 min of 100 nM isoproterenol (16504) (ISO in Ty, 1 mM Ca^2+^) perfusion at 1 Hz stimulation. Fluorescence data is shown as Δ*F*/*F*_0_, where *F*_0_ is the average fluorescence intensity before field stimulation. To evaluate spark characteristics in freshly isolated myocytes, the longitudinal cell axis with 100 nm pixel size records was taken at 1 Hz pace. Analysis was performed using ImageJ software (http://imagej.nih.gov/ij/, NIH, Bethesda, MD, USA) with the SparkMaster plugin [31].

### 2.6. Mitochondrial Membrane Potential in Intact Cardiomyocytes

Freshly isolated cardiomyocyte cells were incubated with 300 nM tetramethylrhodamine ethyl ester perchlorate (TMRE) (T669, Thermo Fisher Scientific, USA) for 30 min at 25°C (in Ty, 1 mM Ca^2+^) [32]. After washing with a fluorophore-free and Ca^2+^-free solution, 2D images (1024 × 1024 pixels, 400 Hz, 1 *μ*m section thickness) were taken using 543 nm excitation and 555-700 nm emission window. Results were normalized to the CTRL group as a percentage. The degree of mitochondrion polarization was then expressed as TMRE intensity per cell. As a negative control, cells were perfused with 0.8 *μ*M cyanide m-chlorophenyl hydrazone (CCCP) (C2759) during 10 min (data not shown).

### 2.7. Mitochondrial Isolation

Heart mitochondrial fractions were obtained according to the method described previously [33]. Briefly, the heart was isolated and placed in an ice-cold buffer (SHE), containing (mM) the following: 250 sucrose, 10 HEPES and 1 EGTA, and pH 7.3. After, only RV tissue was digested for 10 min using 0.12 mg of protease in cold SHE buffer and centrifuged at 800 x g. The homogenates were centrifuged at 10000 x g for 10 min, and then the resulting pellet was suspended in SH buffer containing (mM) the following: 250 sucrose, 10 HEPES, pH 7.3, and free EGTA. Isolated mitochondria were suspended (0.6 mg/ml) in respiration buffer containing (in mM) the following: 125 KCl, 3 KH_2_PO_4_, 10 HEPES, and pH 7.3.

### 2.8. Mitochondrial Function from RV in the PAH Model

For the respiratory studies, 0.1 mg/ml mitochondria in respiratory medium (140 mM K^+^-gluconate, 5 mM KH_2_PO_4_, 2 *μ*g/ml rotenone, 10 mM succinate, 10 mM HEPES pH 7.2) was evaluated during the respiratory states of no-phosphorylation (state 4), phosphorylation (state 3, ADP added), and maximal respiratory activity (uncoupled with FCCP). Oxygen consumption was recorded using high-resolution respirometry (Oroboros Instrument, Innsbruck, Austria) [32].

### 2.9. Ca^2+^ Retention Capacity (CRC)

mPTP opening sensitivity was evaluated in isolated mitochondrial by CRC. Mitochondria were incubated in a medium containing 0.3 *μ*M Calcium Green-5N (C3739, Life Technologies, Carlsbad, CA, US), 10 mM succinate plus rotenone (10 *μ*g/ml), 200 *μ*M ADP, and 0.25 *μ*g Oligomycin A. After 5-minute incubation, 10 *μ*M of Ca^2+^ pulses was added every 3 min and fluorescence was recorded at 488 nm excitation and 500-600 nm emission. After enough Ca^2+^ loading, a massive release of mitochondrial Ca^2+^ indicates mPTP opening. The amount of CaCl_2_ necessary to trigger this enormous Ca^2+^ release was used as an indicator of the susceptibility to mPTP opening due to Ca^2+^ overload [32].

### 2.10. Protein Extraction

50 mg heart tissue was macerated using a Polytron PT1200 E (Kinematica AG, Switzerland) with 500 *μ*l of buffer SHE added with 1 mM phenylmethylsulfonyl fluoride (PMSF, P7626) and 1 mM dithiothreitol (DTT, D0632). Afterwards, the homogenate was centrifuged at 2000 rpm during 10 min at 4°C. Protein was quantified in the supernatant by the Lowry method using bovine serum albumin as standard.

### 2.11. Western Blot

Protein were resolved on SDS-PAGE gel and transferred onto a PVDF membrane, which was incubated with primary antibody. The membrane was washed three times for 10 min with PBS-0.5% Tween 20 and subsequently probed with an HPR-conjugated secondary for 2 hours at 25°C. After washing three times with PBS-0.5% Tween 20 for 10 min, the blots were developed with SuperSignal West Dura Extended Duration Substrate (TG268239, Thermo Fisher Scientific, Waltham, MA, US) and quantified by using a BioSpectrum 415 Image Acquisition System (UVP, Upland, CA, USA). Protein acetylation used 30 *μ*g of isolated mitochondria in a 15% SDS-PAGE gel;to SIRT3 expression, 15 *μ*g of isolated mitochondria was used in a 12% SDS-PAGE gel; electrophoresis and transfer conditions were 105 V, 25°C, 2.5 h, and 300 mA, 4°C, 2 h, respectively, to both. SERCA2, phospholamban (PLB), and D-glyceraldehyde-3-phosphate dehydrogenase (GAPDH) were quantified in the same blot. 40 *μ*g of tissue homogenate was loaded in 12% SDS-PAGE gel. Electrophoresis and transfer conditions were 85 V, 25°C, 2 h, and 350 mA, 25°C, 1.5 h, respectively. Primary antibodies used were as follows: anti-Acetylated-Lysine (9441S, Cell Signaling, Danvers, MA, US) (1 : 1000); anti-SIRT3 (D22A3, Cell Signaling, Danvers, MA, US) (1 : 2000); anti-SERCA (sc-8094, Santa Cruz Biotechnology, MA, US) (1 : 10000); and anti-PLB (sc-21923, Santa Cruz Biotechnology, MA, US) (1 : 1000). Anti-COX4 (4D11-B3-E8, Cell Signaling, Danvers, MA, US) (1 : 2000) [25] and anti-GAPDH (sc-25778, Santa Cruz Biotechnology, MA, US) (1 : 10000) antibodies were used as a loading control.

### 2.12. Real-Time Polymerase Chain Reaction (PCR) Analysis

Total RNA from right ventricles was extracted using TRIzol Reagent (15596026, Invitrogen, Carlsbad, CA, USA). Sample purity was confirmed measuring a 260/280 nm absorbance ratio using a Take3 multivolume plate in a Synergy HT microplate reader (BioTek Instruments, Winooski, USA). SensiFAST cDNA Synthesis Kit (BIO-65053, Bioline, London, UK) was used to reverse-transcribe cDNA from 1 *μ*g of total RNA. qPCR reaction was performed using the SensiFAST SYBR Lo-ROX Kit (BIO-94020, Bioline, London, UK) in a Quant-Studio 3 RT PCR System (Thermo Fisher Scientific, Waltham, TX, USA). Data was analyzed by 2−*ΔΔ*Ct method to estimate mRNA expression from each gene [15, 34]. T4 Oligo (Mexico) synthesized primers. Primer sequences are detailed in Supplementary Table 1.

### 2.13. Oxidative Stress Markers

Free thiol groups were evaluated in 200 *μ*g of isolated mitochondria incubated with 5,5-dithio-bis-(2-nitrobenzoic acid) (DNTB, 300 *μ*M) (D8130), during 10 min at 25°C in darkness. After 10 min centrifugation at 10000 rpm, the supernatant was measured at 412 nm. Protein carbonyl content was assessed in homogenized tissue following assay kit (ab126287, Abcam, UK) instructions. Membrane lipid peroxidation was analyzed by measuring the generation of thiobarbituric acid-reactive substances (TBARS) as previously reported [35]. DNA oxidation was analyzed by ELISA assay according to the kit manufacturer's instructions (ab101245, Abcam, UK). The concentration of 8-hydroxy-20-deoxyguanosine (8-OHdG) was measured using a standard curve and expressed in nanograms per micrograms of DNA [36]. Enzyme activities were evaluated in a homogenized heart tissue. Catalase activity was measured by O_2_ production assessed by an electrode type Clark [36]. Briefly, 50 *μ*g protein was added to phosphate buffer (KH_2_PO_4_ 50 mM, pH 7.8), and the addition of 5 mM H_2_O_2_ (quantified at 240 nm with an extinction coefficient of 43.6 M^−1^ × cm^−1^) started the reaction. Data were expressed as units per milligram of protein (U/mg). Aconitase activity was measured by monitoring *cis*-aconitate synthesis from citrate at 30°C on 240 nm. 150 *μ*g protein was added to 1 ml of medium (100 mM KH_2_PO_4_, 0.01% Triton X-100, 0.6 mM MnCl_2_, 0.2 mM NADP, and 1 mM citrate, pH 7.2). Enzyme activity is expressed as nmol × min^−1^ × mg^−1^, using *cis*-aconitate extinction coefficient (E240 nm = 3.6 mM^−1^ × cm^−1^). Superoxide dismutase activity was measured as previously reported [36]. Briefly, 50 *μ*g protein was added to phosphate buffer (50 mM KH_2_PO_4_, 1 mM EDTA, 10 mM xanthine, 50 *μ*M nitro-blue tetrazolium chloride (NBT), 1 U catalase, 1.5 U Xantina oxidase, pH 7.8). Oxidation of NBT by superoxide anion was measured at 560 nm. To measure Mn-SOD, 3 mM KCN was added. One activity unit was defined as 50% oxidation of NBT. Results were expressed as units per milligram of protein (U/mg).

### 2.14. Immunoprecipitation

As described before [15], isolated mitochondria from right ventricles (1 mg) were solubilized in buffer containing (in mM) the following: 150 NaCl, 1 EGTA, Igepal 1%, 20 Tris-HCl, pH 7.2, and protease inhibitor cocktail (Roche). Afterwards, they were clarified of endogenous IgG and incubated with 2 *μ*g of mouse anti-CypD (ab110324, Abcam, UK), or the isotype IgG as control, 1 hour at 4°C in a rotator. The immunoprecipitation complexes were captured by adding 50% activated slurry of Protein G Sepharose beads (GE) to the solubilized protein and incubated overnight at 4°C in a rotator. Beads were centrifuged and washed thrice. Complexes were eluted in SDS-loading buffer prior to electrophoretic separation and subsequent western blot analysis.

### 2.15. Statistics

Data is presented as the mean ± SEM. Statistical analysis and graphs were performed using GraphPad Prism software (V.5.01; La Jolla, CA, USA). Data were analyzed by one-way ANOVA or two-tailed Student's *t*-test;to compare the groups, Dunn's post hoc test was performed when appropriate. Statistical significance was set at *p* < 0.05.

### 2.16. Study Approval

All procedures performed in animals were supervised and approved by the Internal Committee for Care and Handling of Laboratory Animals of the School of Medicine of the Tecnológico de Monterrey (Protocols no. 2017-006 and 2019-019) and were performed following the Mexican National Laboratory Animal Health Guidelines (NOM 062-ZOO 1999).

## 3. Results

### 3.1. RES Preserves Right Ventricular Function with a Limited Effect on Lung Vasculature

The PAH model requires 28–42 days to develop phenotypic changes in the lungs and heart [37]. Previously, it has been shown that heart alterations caused by MC target the RV and RES treatment improve RV function with a limited protective effect on pulmonary architecture [16]. In addition, rats treated only with RES showed no differences compared to the control animals (without MC) for lung morphological parameters such avessel lumen diameter, number of muscular arteries, luminal occlusion, and RV histological characteristics (Supplemental Figure 1).

### 3.2. RES Prevents Contractility Alterations and Improves Ca^2+^ Handling

**Figure 1:**
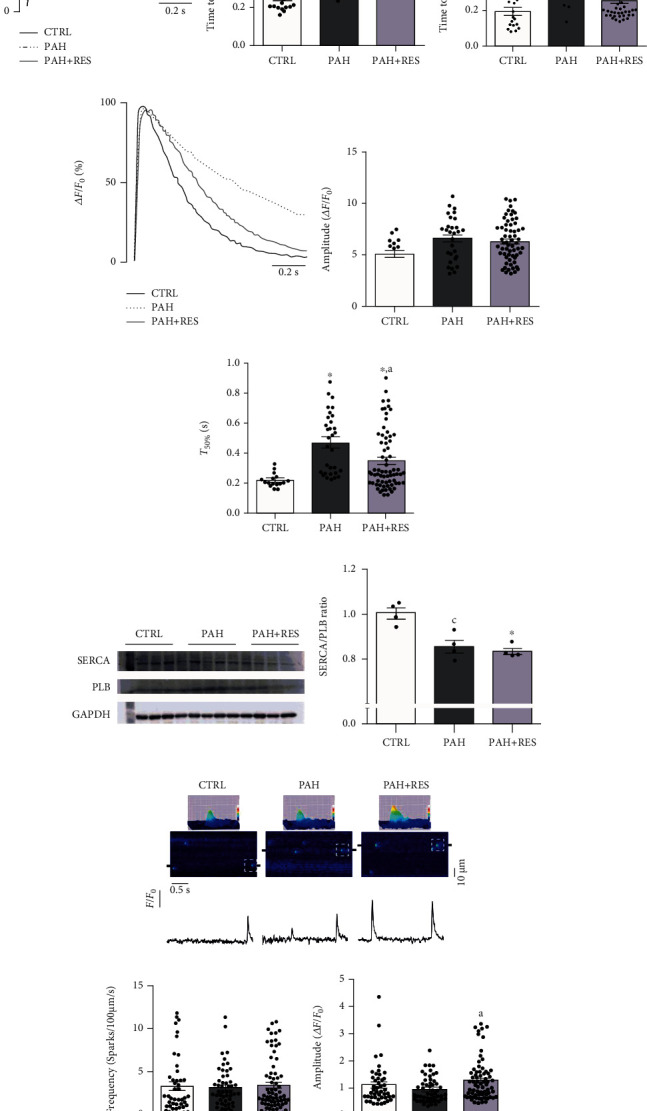
Characterization of cell contraction and Ca^2+^ dynamics in isolated RV myocytes. (a) Representative profile of cellular shortening. Average of time to peak shortening (b) (CTRL: *n* = 20 cells; PAH: *n* = 21 cells; PAH+RES: *n* = 45) and time to 50% relaxation (c) (CTRL: *n* = 18 cells; PAH: *n* = 17 cells; PAH+RES: *n* = 16). (d) Representative florescence profile of Ca^2+^ transient. Pooled data from Ca^2+^ transient amplitude (e) and *T*_50%_ (f) (CTRL: *n* = 16 cells; PAH: *n* = 53 cells; PAH+RES: *n* = 69 cells). (g) Representative images and pooled data of western blot to the SERCA2/PLB ratio; GAPDH was used as a loading control (*n* = 4). (h) Representative line scan images of treated groups; surface plots from selected sparks (doted square) are above; shown below are line profiles from 2 *μ*m regions of the selected spark (black marks in line scan images). Pooled data of Ca^2+^ spark frequency (i) and amplitude (j) (CTRL: *n* = 46 cells, 3 animals; PAH: *n* = 55 cells, 3 animals; PAH+RES: *n* = 71 cells, 5 animals). CTRL (solid black line), pulmonary arterial hypertension (PAH, dotted line), and PAH treated with resveratrol (PAH+RES, solid gray line). CTRL: control; PAH: pulmonary arterial hypertension; PAH+RES: PAH treated with resveratrol. All data are presented as the mean ± SEM. ^∗^*p* < 0.05 vs. CTRL; ^a^*p* < 0.05 vs. PAH, calculated by 1-way ANOVA; ^c^*p* < 0.05 vs. CTRL, calculated by a 2-tailed *t*-test.

RV myocyte function was evaluated by characterizing cell contraction and Ca^2+^ dynamics to assess alterations in excitation-contraction coupling (ECC). Cell shortening was less efficient in the PAH group since time to peak shortening (Figure 1(b)) and half relaxation (Figure 1(c)) were 82% and 41% slower, respectively. RES treatment accelerated the time to peak shortening by 22% in PAH (Figure 1(b)) and maintained the CTRL group time to half relaxation (Figure 1(c)), indicating that RES treatment improved cell relaxation in PAH.

Since intracellular Ca^2+^ dynamics orchestrates cell contractility, transient parameters were characterized. Transient amplitude (Figure 1(e)) did not change between groups. However, in transient decay, the PAH group showed a 1.2-fold slower *T*_50%_ than CTRL, while RES reduced this effect by 26% (Figure 1(f)); however, no significant differences were shown in the SERCA2/PLB ratio between the PAH and PAH+RES groups (Figure 1(g)), indicating that the improved *T*_50%_ might have been due to increased SERCA activity rather than changes in its expression. Gene expression of other Ca^2+^ extrusion mechanisms, Na/Ca^2+^ exchanger (NCX) and mitochondria calcium uniplex (MCU), did not change between groups (NCX: CTRL = 1 ± 0.11, PAH = 1.03 ± 0.15, PAH+RES = 0.86 ± 0.12; MCU: CTRL = 0.88 ± 0.13, PAH = 0.88 ± 0.13, PAH+RES = 0.96 ± 0.15 mRNA/HPRT).

Ca^2+^ spark frequency (Figure 1(i)) and amplitude (Figure 1(j)) were measured as an indicative of RyR activity. No changes were observed between PAH and CTRL groups; thus, RyR activity was not affected. However, the PAH-RES group had a higher spark amplitude than the PAH group (PAH:0.96 ± 0.06Δ*F*/*F*_0_, RES+PAH:1.3 ± 0.09Δ*F*/*F*_0_, *p* < 0.05), suggesting a higher SR Ca^2+^ content.

**Figure 2:**
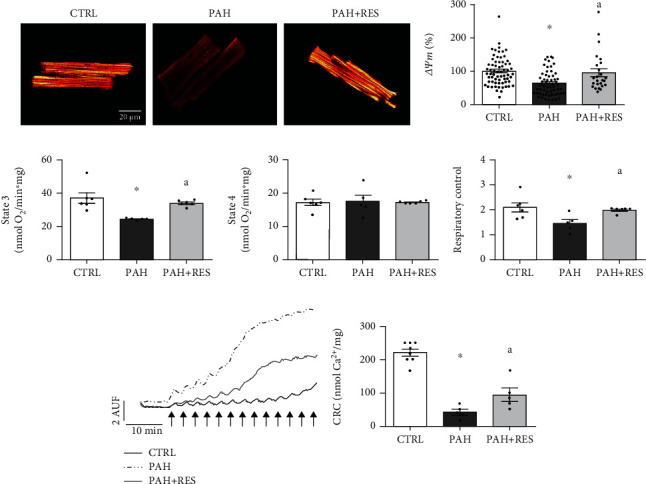
Characterization of mitochondrial function from RV. Representative images (a) and pooled data (b) from ΔΨ*m* in isolated RV myocytes (CTRL: *n* = 66 cells and 3 animals; PAH: *n* = 59 cells and 3 animals; PAH+RES: *n* = 25 cells and 2 animals). Mitochondrial respiratory states (c, d) and respiratory control ratio (e) of isolated mitochondria preparations (CTRL: *n* = 6, PAH: *n* = 5, PAH+RES: *n* = 6). (f) CRC from isolated mitochondria, in left image each arrow represents a 10 nmol CaCl_2_ bolus (CTRL: *n* = 8, PAH: *n* = 5, PAH+RES: *n* = 4; ^a^*p* < 0.05 vs. PAH, calculated by a 2-tailed *t*-test). CTRL: control; PAH: pulmonary arterial hypertension; PAH+RES: PAH treated with resveratrol. All data are presented as the mean ± SEM. ^∗^*p* < 0.05 vs. CTRL; ^a^*p* < 0.05 vs. PAH, calculated by 1-way ANOVA.

RV myocytes were challenged after isoprenaline (ISO, 100 nM) perfusion to induce the *β*-adrenergic response (*β*-AR), a highly energy-dependent state (Supplemental. Fig. 2). All groups were capable to increase Ca^2+^ transient amplitude and to reduce *T*_50%_. However, cell contraction failed to comply in the PAH group, while RES-treated PAH prevented this Ca^2+^ transient and cell contractility mismatch.

### 3.3. RES Prevents Cellular Energetics Failure

Since *β*-AR emphasizes the disruption of excitation-contraction-energetic coupling (ECEC) in PAH, mitochondrial function was evaluated in isolated myocytes and mitochondria from the RV tissue. PAH compromises ATP production by altering mitochondrial functioning, as ΔΨ*m* decreased by 47% in PAH myocytes (*p* < 0.001), while in RES-treated PAH, the control level was sustained (95.4 ± 11.6%) (Figure 2(b)). RV mitochondria from the PAH group also showed a 26% reduction in respiratory activity during state 3 of respiration (Figure 2(c)) with no change in basal respiratory activity in state 4 (Figure 2(d)), thus decreasing respiratory control ratio (CTRL: 2.1 ± 0.19; PAH: 1.5 ± 0.15) (Figure 2(e)). In the PAH-RES group, mitochondria maintained the respiratory control ratio (2 ± 0.04) (Figure 2(e)) by preserving the phosphorylation response (CTRL: 36.93 ± 3.24 nmol O_2_/min∙mg; PAH+RES: 33.89 ± 0.69 nmol O_2_/min∙mg) (Figure 2(c)). Isolated mitochondria from the PAH group also showed increased mPTP, demonstrated by an 81% decrease in Ca^2+^ retention capacity (CRC), while RES treatment produced a 2.5-fold decrease in mitochondrial fragility in the PAH-RES group (Figure 2(f)). The enhanced permeability was a consequence of mPTP opening since the effect is inhibited by CsA, a potent inhibitor of the mPTP (mean ± SEM: CTRL = 216.7 ± 33.33 nmol Ca^2+^/mg, PAH = 186.1 ± 45.24 nmol Ca^2+^/mg, PAH+RES = 186.7 ± 54.52 nmol Ca^2+^/mg; *p* = 0.3831; *n*: 7, 6, and 5 mitochondrial preparations, respectively).

**Figure 3:**
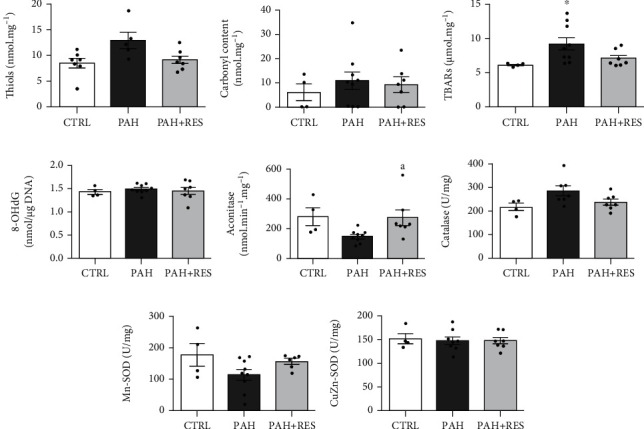
Oxidative stress markers in RV. Pooled data from free thiol groups in isolated mitochondria (a). Average of protein carbonylation (b), TBARS (c), and 8-OHdG/total DNA ratio in RV tissue (d). Enzymatic activity of aconitase (e), catalase (f), manganese superoxide dismutase (Mn-SOD) (g), and copper-zinc superoxide dismutase (CuZn-SOD) (h). Control (CTRL: *n* = 4-7); pulmonary arterial hypertension (PAH, *n* = 5-9); PAH treated with resveratrol (PAH+RES, *n* = 6-7). All data are presented as the mean ± SEM. ^∗^*p* < 0.05 vs. CTRL; ^a^*p* < 0.05 vs. PAH, calculated by 1-way ANOVA.

The involvement of oxidative stress in mitochondria permeability transitioning was also evaluated. As shown in Figure 3(b), there was an increase in membrane peroxidation concomitant with 48% decrease in aconitase activity in the PAH group indicating an increased oxidant environment within the mitochondria. However, other antioxidant enzymes and oxidation in protein or DNA did not change in the PAH group (Figure 3). RES treatment maintained the same level of aconitase activity as CTRL (97%; Figure 3(e)) and increased mitochondrial superoxide dismutase (SOD) by 36% compared to the PAH group (Figure 3(g)), which is a direct target of SIRT3.

### 3.4. RES Decreases the Acetylation of CypD

**Figure 4:**
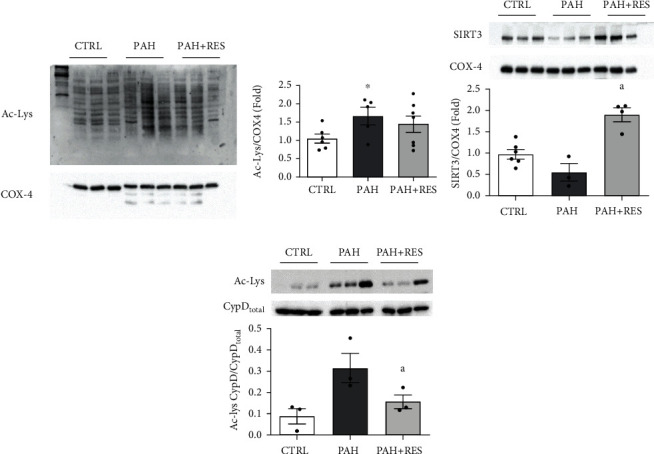
Protein acetylation and SIRT3 expression in RV. Representative image and pooled data of western blot to acetylated lysine (Ac-Lys) profile from isolated mitochondria (a) (CTRL: *n* = 5, PAH: *n* = 4, PAH+RES: *n* = 6). ^∗^*p* < 0.05 vs. CTRL unpaired 2-tailed *t*-test. (b) Representative images and pooled data of western blot from isolated mitochondria against SIRT3; cytochrome c oxidase subunit 4 (COX-4) was used as a loading control (CTRL: *n* = 6, PAH: *n* = 3, PAH+RES: *n* = 4). (c) Immunoprecipitation (IP) of cyclophilin D (CypD) followed by immunoblot analysis against Ac-Lys; acetylation signal was normalized to total CypD (CTRL: *n* = 3, PAH: *n* = 3, PAH+RES: *n* = 3). Control (CTRL); pulmonary arterial hypertension (PAH); PAH treated with resveratrol (PAH+RES). All data are presented as the mean ± SEM. ^∗^*p* < 0.05 vs. CTRL, ^a^*p* < 0.05 vs. PAH, calculated by 1-way ANOVA.

To evaluate the extent of sirtuin activation by RES, the mitochondrial protein acetylation profile, SIRT3 expression, and acetylation of CypD were assessed. In Figure 4(a), the protein acetylation profiles of isolated mitochondria show a 59% increase in acetylated proteins in the PAH group, including a threefold increase in CypD acetylation (Figure 4(c)). Protein acetylation was decreased by 13% in the PAH-RES group (*p* = 0.5303 vs. PAH); however, SIRT3 was 96% overexpressed (Figure 4(b)). Importantly, CypD was a critical component of mitochondria permeability transitioning, as shown by the significant decrease of 51% (*p* = 0.0581, vs. PAH) in acetylation in the PAH-RES group (Figure 4(c)), indicating the importance of SIRT3 in reducing mitochondrial permeability transitioning. There were no significant between-group differences in SIRT1 gene expression (CTRL = 0.89 ± 0.08; PAH = 0.96 ± 0.12; PAH+RES = 0.82 ± 0.09mRNA/HPRT), while SIRT5 decreased in both the PAH and PAH+RES groups (CTRL = 0.92 ± 0.11; PAH = 0.43 ± 0.08; PAH+RES = 0.45 ± 0.071mRNA/HPRT).

## 4. Discussion

The cardiovascular protective actions of RES in PAH have been reported in RV function [16, 38]; however, the mechanisms involved have not been fully described. According to our results, PAH causes an energetic dysfunction in the RV myocyte by promoting the opening of the mPTP due to CypD hyperacetylation. The decreased energy supply impairs the highly energy-demanding processes involved in myocyte functions, such as cellular relaxation, by hampering SERCA activity and delaying myofibrils' unbinding. Treating this model with RES stimulates and overexpresses SIRT3, which prevents mPTP opening by acting directly on CypD deacetylation. Preserving cellular energetics also preserves myocyte contraction and relaxation.

### 4.1. RES Improves Cell Relaxation and SERCA Activity

Intracellular Ca^2+^ oscillations orchestrate cell contraction-relaxation cycle during Ca^2+^ transient, since cytosolic Ca^2+^ interacts with troponin C allowing actin-myosin interaction.

RV myocyte functioning was evaluated using confocal microscopy by assessing intracellular Ca^2+^ signaling and cell shortening. Characterization of the cellular contraction-relaxation cycle showed that PAH has a negative impact on the overall contraction-relaxation dynamic. Regardless, no alterations in SR Ca^2+^ release synchronicity during Ca^2+^ transient; it takes longer to accomplish maximal shortening. Treating PAH with RES decreased time to maximal shortening possibly by increased myofilament Ca^2+^ sensitivity [39, 40]; however, it does not fully normalize this parameter. Interestingly, relaxation dynamics showed the most significant changes. PAH considerably prolongs cell relaxation, while PAH under RES treatment keeps the relaxation rate similar to the CTRL group, a finding that has not been previously reported.

Accordingly, major alteration in intracellular Ca^2+^ transient caused by PAH was a decreased SERCA activity caused by a decreased SERCA/PLB ratio. However, both increased [41] and decreased [42] SERCA activities have been reported, as well as no change in SERCA and PLB expression [41]. Although RES treatment did not change the SERCA/PLB ratio, it did increase intracellular Ca^2+^ removal, thus SERCA activity.

This behavior was replicated after stimulating the *β*-AR, a highly energy-demanding condition [43, 44]. PAH showed the highest transient amplitude but the lowest cell shortening, while RES preserved the proportionality of Ca^2+^ released and cell contraction.

This effect might be related to a mismatch in the ECEC since it has been documented that creatine kinase (CK) expression is diminished in failing RV caused by PAH due to a decreased ATP supply [21, 45, 46]. Failing to meet the energetic demand, myofilament cross-bridge cycling is inhibited in finding shorter sarcomere lengths [21], compromising myocyte function. In this regard, the preservation of ATP production by RES preserves CK activity and an efficient ATP supply to SERCA, displaying improved cell contraction and relaxation.

Additionally, PAH did not modify Ca^2+^ spark frequency, indicating intact RyR activity [47], unlike previous reports [41, 42]. RES treatment also showed no changes in spark frequency and amplitude.

### 4.2. RES Preserves Mitochondrial Functioning and Integrity

Since major alterations caused by PAH reside among highly energy-dependent mechanisms, cell excitation and contraction must be tightly linked to the energy supply to ensure the proper functioning of the myocyte as a whole in an ECEC process. Since mitochondrial dysfunction has been identified as one of the mechanisms underlying heart failure [18, 19, 22, 46], mitochondrial activity was evaluated in this model. In isolated myocytes, PAH compromises ATP production by decreasing ΔΨ*m*; the electrochemical force needed for ATP production, decreasing the oxidative phosphorylation rate (state 3). Decreased phosphorylation response have been reported in RV by PAH [48–50]. The cardioprotective effects of RES on mitochondria have been described in several models [51–53]. Similarly, RES treatment preserved ΔΨ*m* and respiratory chain activity in PAH. Even though the results obtained by mitochondrial respiration and fragility are evident when stimulating complex II, these same aspects have yet to be evaluated under NADH-dependent respiration.

Mitochondrial function preservation by RES in PAH could be related to decrease mitochondrial fragility. The loss of mitochondrial integrity by mPTP formation has been described as a main contributor to ventricular dysfunction in the right and left ventricles [22, 54, 55]. Most importantly, preventing mPTP opening using CsA has been shown to reduce RV dysfunction in PAH [22]. Mitochondrial Ca^2+^ overload in PAH, complemented by an increased oxidative environment within the mitochondria, makes them more prone to permeability transition [54, 56]; however, unlike other reports [57], no critical signs of proteins being modified by oxidative stress were observed, indicating that the antioxidant system is capable of managing oxidative stress caused by PAH in this model.

Although evident impairment in ΔΨ*m* was found in this study, outright evidence from reduced cellular bioenergetics in this PAH model would directly measure ATP levels and evaluate the phosphocreatine system. However, it is well established that the ATP phosphorylation is particularly sensitive to decreases of mitochondrial membrane potential; for every 14 mV decrease in proton-motive force (equivalent to ΔΨ*m*), the ATP/ADP ratio decreases by 10-fold [58]. Also, the mitochondrial membrane potential has been described as a crucial factor to generate rotational torque by the *F*_0_ nanomotor [59, 60]. Thus, a decrease in this electrochemical force may impede ATP production. In this regard, the integrity of ΔΨ*m* in PAH+RES RV myocytes suggests a capable, energetic system to sustain the cardiac ECC.

### 4.3. RES Promotes the Deacetylation of CypD by SIRT3 Overexpression

One of the mechanisms through which RES bestows its protective effects is the activation of deacetylases. In pulmonary arterial smooth muscle cells (PASMC), the protective effect of RES has been linked to expression and activity modulation of the cytosolic deacetylase SIRT1 [61, 62]. Similarly, an increase in acetylated proteins was found in PAH RV myocytes [16, 38], and the cardiac protection of RES through SIRT1 activation has been reported in different conditions [38, 63–65]. Since PAH's effect is on mitochondrial function and activity, the expression of SIRT3 and mitochondrial acetylome were evaluated to determine the extent of its involvement.

**Figure 5:**
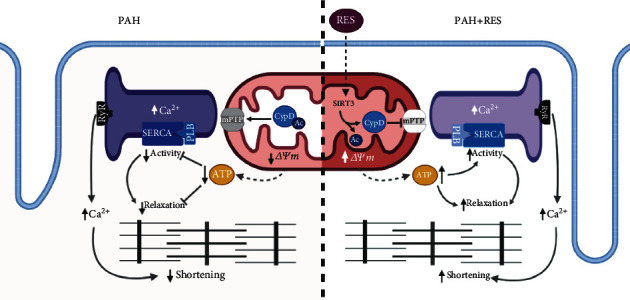
Proposed mechanism of RES cardioprotection. Resveratrol stimulates SIRT3 activation and expression, which deacetylates CypD preventing mPTP formation and preserving ΔΨ*m*, thus increasing ATP synthesis. The preserved mitochondrial function promotes a high-energy demand process to occur, such as cellular relaxation and SERCA activity, preventing excitation-contraction-energetic coupling mismatch. RES: resveratrol; LTCC: L-type Ca^2+^ channel; RyR: ryanodine receptor; SERCA2: sarco-endoplasmic reticulum Ca^2+^-ATPase; PLB: phospholamban; mPTP: mitochondrial permeability and transition pore; CypD: cyclophilin D; Ac: acetyl-group; SIRT3: sirtuin 3; ΔΨ*m*: mitochondrial membrane potential. Created with http://BioRender.com.

The mitochondrial deacetylase SIRT3, which regulates mitochondrial function [66–69], is modulated by RES [12], and the rs11246020 polymorphism, associated with a ~30% loss of function, has been found to be overrepresented in patients with idiopathic PAH [25]. PAH showed an increase in the protein acetylation profile from mitochondria, similar to reports in PASMC [25]. RES treatment increased SIRT3 expression almost 3.5-fold; however, the acetylation profile did not decrease significantly from PAH levels. Regardless of this discrepancy, an important direct target of SIRT3, cyclophilin D (CypD), decreased by almost half its hyperacetylation, diminishing the mitochondria's proneness to mPTP opening [15, 23], thus preserving the cellular energetic state (Figure 5).

Furthermore, SIRT3 activity has been linked to the prevention of myocardial dysfunction. Treatment with honokiol, a potent SIRT3 activator, prevents and reverses ventricular hypertrophy [70] and preserves mitochondrial integrity and energetic capability [70, 71]. The mechanisms involved still need to be elucidated; however, it has been found that SIRT3 prevents mitochondrial Ca^2+^ overload by regulating mitochondrial Ca^2+^ uptake, regulating the expression of mitochondrial Ca^2+^ uniporter and its main regulator MICU1. These data are relevant because it was established that MCU and the MICU1 promoter are regulated by SIRT3-dependent histone acetylation [72]. However, no change in MCU gene expression was found in this model.

While SIRT3 activity has been identified as being involved in cardiovascular protection [73–77], it is not the only mechanism through which RES acts as a cardioprotective agent. Other sirtuins and enzymes, such as SIRT1, SIRT5, and AMPK [5, 6, 78], contribute to modulating the activation or repression of a wide range of cellular components [5, 6, 78]. Furthermore, PAH pathogenesis is not caused by a single modification; according to the widely accepted “multiple-hit” hypothesis: inflammation, genetic determinants, and environmental factors are needed to develop the disease [79, 80].

Another important mechanism involved in modulating mitochondrial functioning that we did not explore thoroughly in this study is mitochondrial dynamics. The deacetylation of OPA1 by SIRT3 has been described as a regulator of mitochondrial dynamics and plays a role in maintaining a competent mitochondrial population in the heart when it is under pathological stress [81]. While previous reports did not find changes in the gene expression of proteins involved in mitochondrial dynamics and biogenesis in PAH [82], it has been reported that RES promotes mitochondrial fission [83]. This phenomenon is needed to preserve mitochondrial function [84] and may be involved in ΔΨ*m* preservation by RES in this model. However, the implications of mitochondrial dynamics in the progression of RV failure still need to be addressed; this phenomenon could be an interesting area of research in the future to elucidate new therapeutic targets in PAH.

Although we found a strong relationship between preservation of mitochondrial function and CypD acetylation via SIRT3, loss and gain of function experiments can assess its definitive role in this model. The availability of deficient-SIRT3 mice faces the challenge of PHA model development, since it has been reported that mouse PAH models may not develop pulmonary hypertension, RV hypertrophy, and RV failure regardless pulmonary artery remodeling; besides, some of the models show spontaneous reverse of pulmonary hypertension once the inductor is not present [85, 86].

### 4.4. Clinical Implications

This study suggests that mitochondrial protection and cellular energetic preservation could be possible therapeutic targets to improve the PAH phenotype. The currently approved PAH medications decrease vascular remodeling by restoring the balance between vasoactive and vasodilator mediators [87]. However, researchers have begun to focus on mitochondrial disorders to develop new strategies. Molecules that prevent and reverse mitochondrial bioenergetic abnormalities in PASMC are being explored due to their ability to promote apoptosis, thus decreasing pulmonary vascular remodeling [88–91] and, in RV fibroblasts, reducing cardiac fibrosis in PAH [92]. Unfortunately, cardioprotective effects on RV muscle cells have not been explored. Although RES' usefulness in treating cardiovascular diseases has been observed as an overall beneficial effect in diverse clinical trials, further research is needed to ensure sustained effectiveness in patients since its major disadvantage is its poor bioavailability [93]. No published studies have evaluated RES use in PAH patients. Finally, ECEC disruption is a phenomenon found in the right and left heart failure. Describing the underlying mechanism and identifying shared targets may be useful to recognize therapeutic strategies that can help to improve heart failure prognosis and care.

## 5. Conclusions

The present work assessed the alterations in RV myocytes caused by PAH and RES' protection. For the first time, we showed that the development of PAH causes a mismatch in the ECEC: compromised mitochondrial functioning and altered ATP synthesis resulted in deficient cell relaxation and disrupted cell contraction. RES protects mitochondria integrity by decreasing CypD hyperacetylation and increasing SIRT3 expression and activity, preventing mPTP opening, and preserving the ΔΨ*m*. Maintaining cellular energetics preserves ECEC, ensuring the proper functioning of the myocyte as a whole. Understanding the mechanisms involved in the protection that RES confers on the RV in PAH could facilitate the development of new adjuvant therapies that improve the daily lives of PAH patients.

## Data Availability

The data used to support the findings of this study are available from the corresponding author upon request.

## Supplementary Materials

Supplementary Materials Supplemental Table 1: qPCR primer sequences hypoxanthine phosphoribosyltransferase 1 (HPRT), Na^+^/Ca^2+^ exchanger (NCX), sirtuin 1 (Sirt1), sirtuin 5 (Sirt5), and mitochondria calcium uniplex (MCU). HPRT was used as a housekeeping gene. Supplementary Figure 1: heart and lung structural comparison between control animals and RES-treated control animals. Pooled data of muscularized arteries (A), luminal diameter (B), and occlusion (C) in the lung tissue (H&E, 10x). Bar graph depicting the myocyte area (D) in RV. Data was normalized to CTRL mean values. All data are presented as the mean ± SEM. ^∗^*p* < 0.05 vs. CTRL calculated by a *t*-test followed by the Mann–Whitney test. Supplementary Figure 2: characterization of RV myocytes Ca^2+^ transient in isolated RV myocytes. (A) Representative line scan images of Ca^2+^ transient before (baseline) and after *β*-adrenergic stimulation with 100 nM isoproterenol (ISO). Pooled data of Ca^2+^ transient amplitude (B), time to 50% decay (*T*_50%_, C), and time to peak (D) (CTRL:*n* = 115.48cells and 4 animals; PAH:*n* = 51.36cells and 3-4 animals; PAH+RES:*n* = 68.37cells and 4 animals; baseline and ISO, respectively). (E) Percentage of maximal shortening before and after *β*-adrenergic stimulation (CTRL:*n* = 40.8cells and 1-4 animals; PAH:*n* = 26.35cells and 3-4 animals; PAH+RES:*n* = 54.36cells and 4 animals; baseline and ISO, respectively). Records were taken under 1 Hz pace. CTRL: control; PAH: pulmonary arterial hypertension; PAH+RES: PAH treated with resveratrol. All data are presented as the mean ± SEM, unless otherwise stated. ^∗^*p* < 0.05 vs. respective CTRL, ^a^*p* < 0.05 vs. respective PAH, and ^b^*p* < 0.05 vs. respective basal, calculated by 1-way ANOVA.
