# Supplementary material for: Resveratrol Prevents Right Ventricle Dysfunction, Calcium Mishandling, and Energetic Failure via SIRT3 Stimulation in Pulmonary Arterial Hypertension
Source: Oxid Med Cell Longev. 2021 Jun 20;2021:9912434. doi: 10.1155/2021/9912434 (PMC8238598; doi:10.1155/2021/9912434)
Supplement: Supplementary Materials — Supplemental Table 1: qPCR primer sequences hypoxanthine phosphoribosyltransferase 1 (HPRT), Na+/Ca2+ exchanger (NCX), sirtuin 1 (Sirt1), sirtuin 5 (Sirt5), and mitochondria calcium uniplex (MCU). HPRT was used as a housekeeping gene. Supplementary Figure 1: heart and lung structural comparison between control animals and RES-treated control animals. Pooled data of muscularized arteries (A), luminal diameter (B), and occlusion (C) in the lung tissue (H&E, 10x). Bar graph depicting the myocyte area (D) in RV. Data was normalized to CTRL mean values. All data are presented as the mean ± SEM. ∗p < 0.05 vs. CTRL calculated by a t-test followed by the Mann–Whitney test. Supplementary Figure 2: characterization of RV myocytes Ca2+ transient in isolated RV myocytes. (A) Representative line scan images of Ca2+ transient before (baseline) and after β-adrenergic stimulation with 100 nM isoproterenol (ISO). Pooled data of Ca2+ transient amplitude (B), time to 50% decay (T50%, C), and time to peak (D) (CTRL:n = 115.48cells and 4 animals; PAH:n = 51.36cells and 3-4 animals; PAH+RES:n = 68.37cells and 4 animals; baseline and ISO, respectively). (E) Percentage of maximal shortening before and after β-adrenergic stimulation (CTRL:n = 40.8cells and 1-4 animals; PAH:n = 26.35cells and 3-4 animals; PAH+RES:n = 54.36cells and 4 animals; baseline and ISO, respectively). Records were taken under 1 Hz pace. CTRL: control; PAH: pulmonary arterial hypertension; PAH+RES: PAH treated with resveratrol. All data are presented as the mean ± SEM, unless otherwise stated. ∗p < 0.05 vs. respective CTRL, ap < 0.05 vs. respective PAH, and bp < 0.05 vs. respective basal, calculated by 1-way ANOVA. [file 9912434.f1.docx]

**Supplemental Table 1. qPCR primers sequences**

| **Gene name** | **Forward primer 5’-3’** | **Reverse primer 5’-3’** |
| --- | --- | --- |
| **HPRT** | \| CGTGATTAGTGATGATGAACC \| \| --- \| | \| GAGCAAGTCTTTCAGTCCT \| \| --- \| |
| **NCX** | GATGAGTGAGAAGAAAGCCCTGT | ACGGTAGAGGGAATCGGATGA |
| **Sirt1** | GAACCTCTGCCTCATCTA | \| TACTCGCCACCTAACCTA \| \| --- \| |
| **Sirt5** | \| GGGCTGGTGTTAGTGCG \| \| --- \| | \| GGTTGGGTTCCTTGTTCC \| \| --- \| |
| **MCU** | GTTGTGCCCTCTGATGAC | GAGTCCGAGATAGGCTTGA |

Hypoxanthine Phosphoribosyltransferase 1 (HPRT), Na^+^/Ca^2+^ exchanger (NCX), sirtuins 1 (Sirt1), sirtuins 5 (Sirt5), mitochondria calcium uniplex (MCU). HPRT was used as housekeeping.

**Supplementary Figure 1. Heart and lung structural comparison between control animals and RES-treated control animals.** Pooled data of muscularized arteries (A), luminal diameter (B) and occlusion (C) in lung tissue (H&E, 10x). Bar graph depicting the myocyte area (D) in RV. Data was normalized to CTRL mean values. All data are present as mean ± SEM. *p<0.05 vs CTRL calculated by t-test followed by Mann-Whitney test.

**Supplementary Figure 2. Characterization of RV myocytes Ca^2+^ transient in isolated RV myocytes.** (A) Representative line scan images of Ca^2+^ transient before (baseline) and after β-adrenergic stimulation with 100 nM isoproterenol (ISO). Pooled data of Ca^2+^ transient amplitude (B), time to 50% decay (T_50%_, C), and time to peak (D) (CTRL n=115,48 cells and 4 animals; PAH n=51,36 cells and 3-4 animals; PAH+RES n=68,37 cells and 4 animals; baseline and ISO respectively). (E) Percentage of maximal shortening before and after β-adrenergic stimulation (CTRL n=40,8 cells and 1-4 animals; PAH n=26,35 cells and 3-4 animals; PAH+RES n=54,36 cells and 4 animals; baseline and ISO respectively). Records were taken under 1 Hz pace. Control (CTRL); pulmonary arterial hypertension (PAH); PAH treated with resveratrol (PAH+RES). All data are present as mean ± SEM, unless otherwise stated. **p*<0.05 vs respective CTRL, ^a^*p*<0.05 vs respective PAH, ^b^*p*<0.05 vs respective basal, calculated by 1-way ANOVA.
